# Surgical removal of a long-retained vaginal foreign body with extensive cicatricial stenosis: a case report

**DOI:** 10.1093/jscr/rjag897

**Published:** 2026-10-07

**Authors:** Hajime Morimoto, Takashi Shibata, Shigeki Nishikawa, Yasunori Fukuoka, Hiroki Kato

**Affiliations:** Department of Obstetrics and Gynecology, Takatsuki General Hospital, 1-3-13 Kosobe-cho, Takatsuki, Osaka 569-1192, Japan; Department of Obstetrics and Gynecology, Takatsuki General Hospital, 1-3-13 Kosobe-cho, Takatsuki, Osaka 569-1192, Japan; Department of Obstetrics and Gynecology, Takatsuki General Hospital, 1-3-13 Kosobe-cho, Takatsuki, Osaka 569-1192, Japan; Department of Obstetrics and Gynecology, Takatsuki General Hospital, 1-3-13 Kosobe-cho, Takatsuki, Osaka 569-1192, Japan; Department of Obstetrics and Gynecology, Takatsuki General Hospital, 1-3-13 Kosobe-cho, Takatsuki, Osaka 569-1192, Japan

**Keywords:** vaginal foreign body, vaginal stenosis, foreign body removal, scar tissue, surgical technique

## Abstract

Long-retained vaginal foreign bodies may cause severe vaginal stenosis, making identification and removal difficult. A multiparous woman in her 60s underwent magnetic resonance imaging for pyuria that was refractory to treatment, revealing a vaginal foreign body. The vaginal canal was almost completely obstructed ~3 cm from the introitus. The patient disclosed that a plastic end cap from a broom handle had remained in her vagina for ~15 years. During transvaginal removal, the palpable induration initially presumed to be the foreign body was found to be thick, extensive scar tissue, with the actual foreign body being located deeper than anticipated. The boundary between the scar tissue and the normal vaginal wall was indistinct, so careful stepwise dissection was required to reach and remove the foreign body while avoiding bladder and rectal injury. Long-retained vaginal foreign bodies may be surrounded by unexpectedly extensive scar tissue, requiring cautious surgical dissection.

## Introduction

Long-term retention of vaginal foreign bodies may result in complications such as infection, ulceration, necrosis, fistula formation, and vaginal stenosis [1–3]. We encountered a case in which a foreign body had been retained in the patient’s vagina for 15 years, resulting in marked vaginal stenosis. Reports detailing removal techniques in cases with severe vaginal stenosis are limited. We describe the surgical approach and intraoperative considerations for foreign body removal.

## Case report

A postmenopausal woman in her 60s with one prior delivery underwent magnetic resonance imaging (MRI) for refractory pyuria, revealing a vaginal foreign body (Fig. 1A). Her only symptom was mild discomfort during urination. Speculum examination revealed a small amount of malodorous, purulent vaginal discharge, and the vaginal canal was almost completely obstructed by severe stenosis ~3 cm from the introitus (Fig. 2). Transvaginal ultrasonography demonstrated a hyperechoic lesion with posterior acoustic shadowing, which was considered to reflect either a foreign body or calcification (Fig. 1B). The vaginal wall at the stenotic segment was extremely firm, and palpation suggested that the foreign body itself was being felt through the vaginal wall. When a probe was inserted into a small pinhole opening in the stenotic area, a small amount of pus drained out. The patient disclosed that more than 15 years earlier, she had inserted the handle of a broom into her vagina and the plastic cap at its tip had detached and remained there. Removal was considered necessary to improve symptoms and to prevent future vesicovaginal or rectovaginal fistula formation. Based on the preoperative examination, we expected that incising the stenotic tissue would expose the foreign body and allow easy removal (Fig. 3A). However, intraoperatively, the scarred stenotic segment was found to be much thicker and more extensive than anticipated, and the foreign body itself was difficult to identify (Fig. 3B). The surgical procedure was performed as follows:

**Figure 1:**
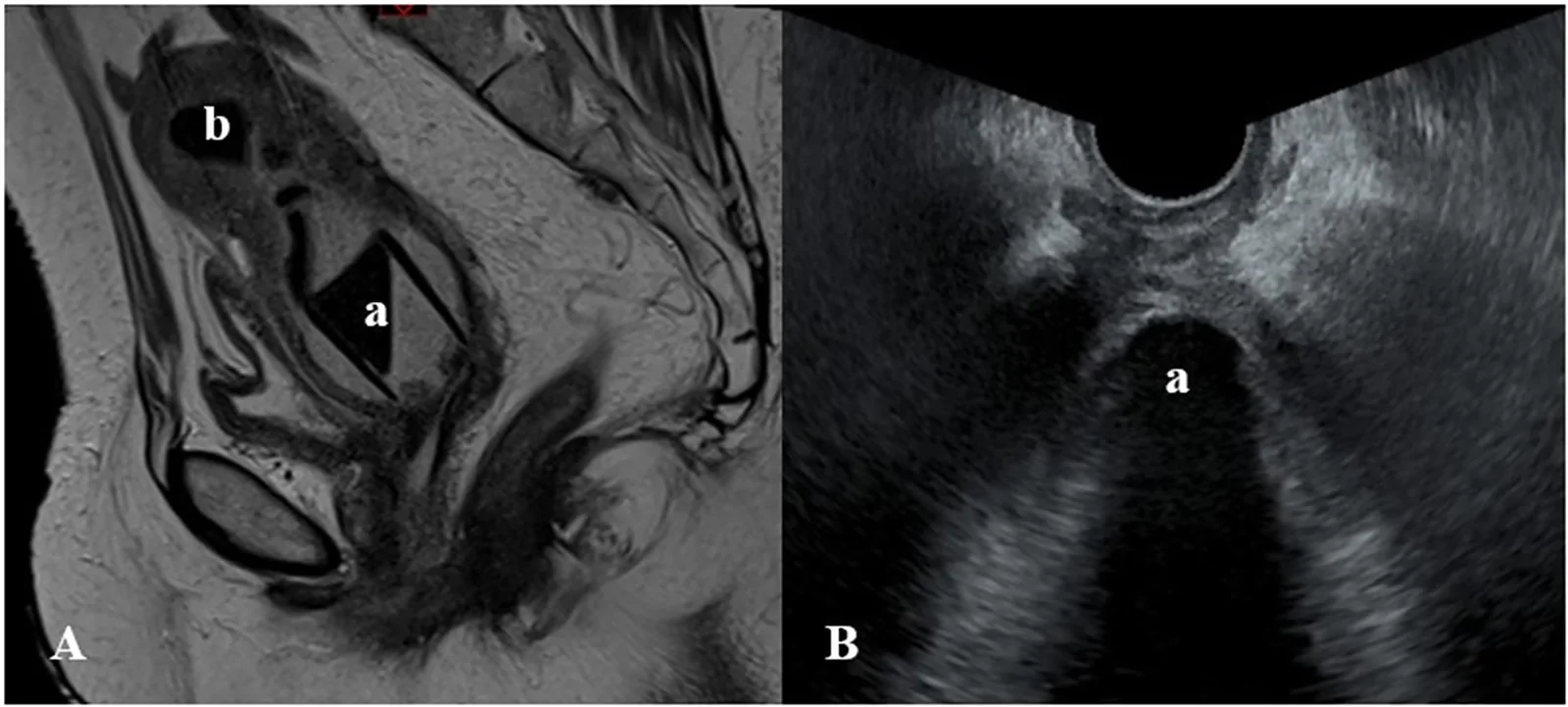
Preoperative imaging findings; (A) sagittal T2-weighted magnetic resonance image showing a vaginal foreign body containing internal fluid, which appeared to extend toward the cervical canal; (B) transvaginal ultrasonography showing a hyperechoic lesion with posterior acoustic shadowing, suggestive of a foreign body or calcification; (a) vaginal foreign body; (b) uterine cavity.

**Figure 2:**
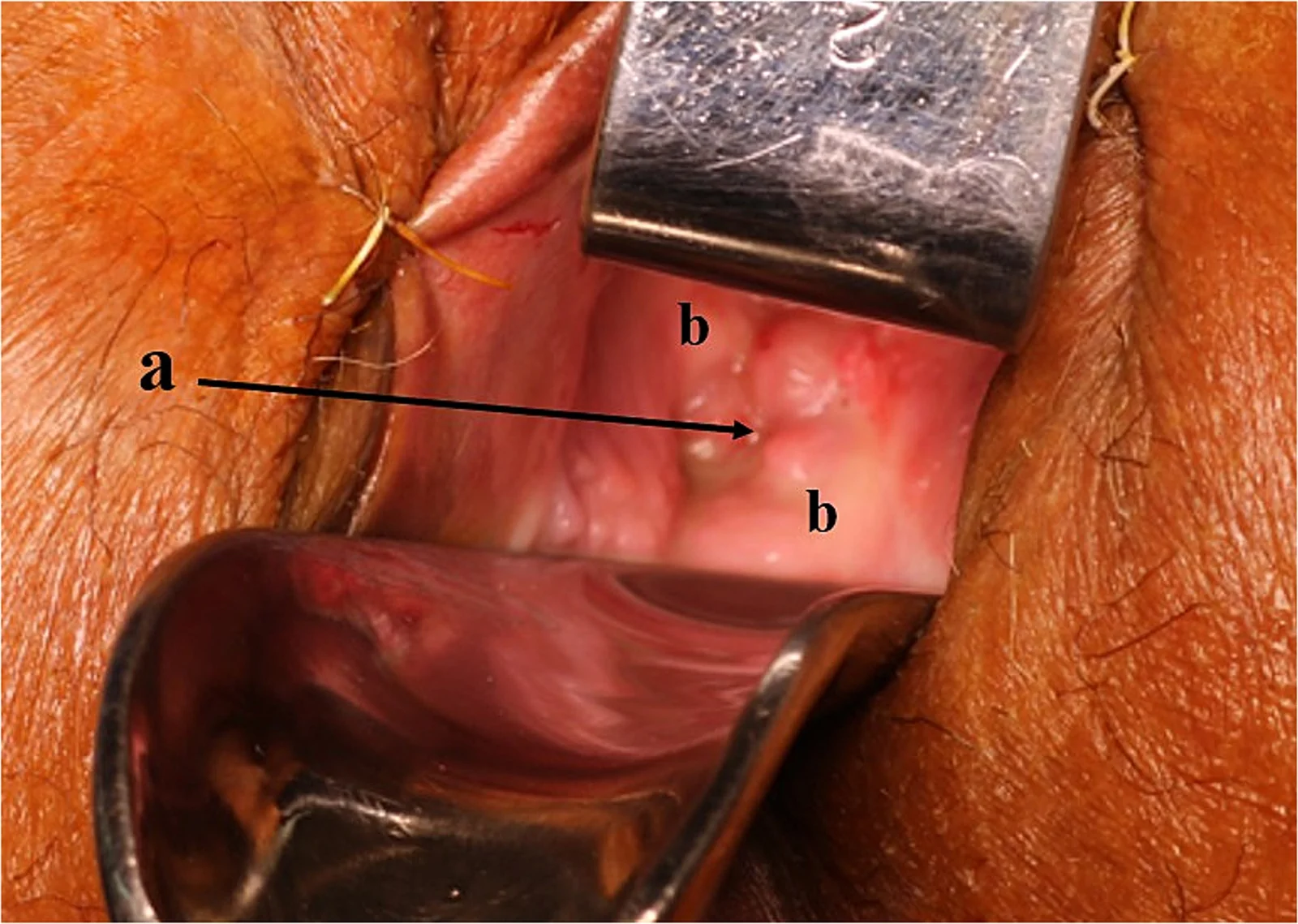
Stenotic vaginal canal; at ~3 cm from the introitus, the vaginal canal was stenotic and nearly completely occluded; (a) pinhole-like opening; (b) scarred area.

**Figure 3:**
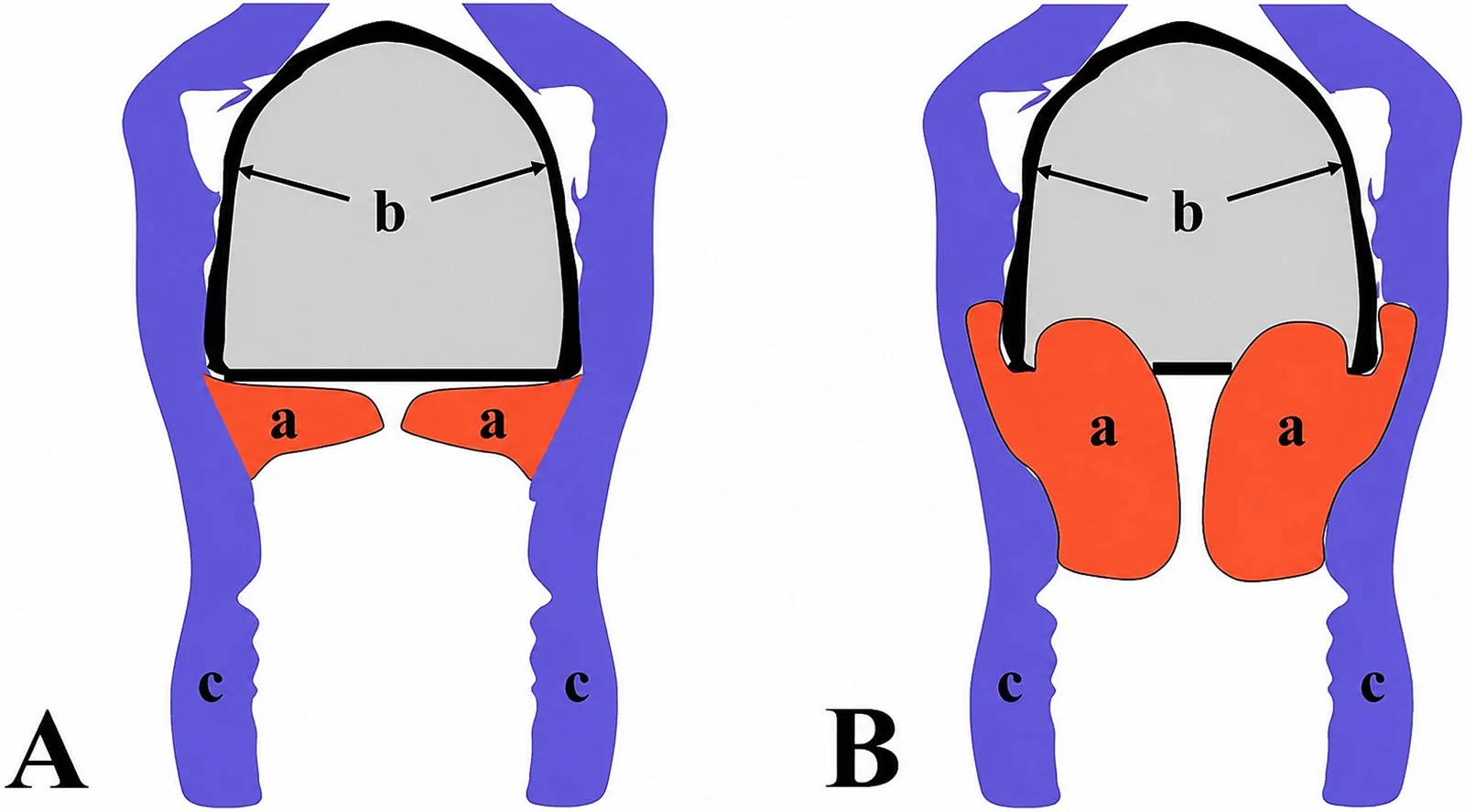
Positional relationship between the foreign body and the stenotic segment caused by scar tissue; (A) preoperative assumption: the induration at the stenotic segment was thought to represent direct palpation of the foreign body itself; (B) actual condition: the stenotic segment was much more extensive than anticipated; (a) scar-related vaginal stenotic segment; (b) vaginal foreign body; (c) normal vaginal wall.

To facilitate detection of any intraoperative bladder injury, 5 ml of indigo carmine was instilled into the bladder. A perineal incision was made to secure the operative field and extended along the posterior vaginal wall toward the stenotic segment. Through this incision, we assessed the anatomical relationship between the stenotic segment and the rectum with the aid of digital rectal examination. If the stenotic tissue had been adherent to the rectum, rectovaginal dissection would have been initiated from this incision to identify a safe dissection plane and minimize the risk of rectal injury during separation of the stenotic tissue from the surrounding tissues. However, a sufficient distance between the stenotic segment and the rectal wall was confirmed, and further rectovaginal dissection was unnecessary.

The pinhole opening was dilated using Hegar dilators to facilitate grasping of the stenotic tissue. The hardened tissue was grasped with Kocher clamps and traction was applied toward the introitus. Although the boundary between the stenotic tissue and the normal vaginal wall was indistinct, a circumferential incision was carefully made in the region of the presumed boundary. From this incision, the indurated stenotic tissue was carefully and progressively separated from the normal vaginal wall by sharp dissection with Cooper scissors and blunt dissection with gauze (Fig. 4). A longitudinal incision was then made in the stenotic tissue exposed by the dissection in an attempt to identify the foreign body.

**Figure 4:**
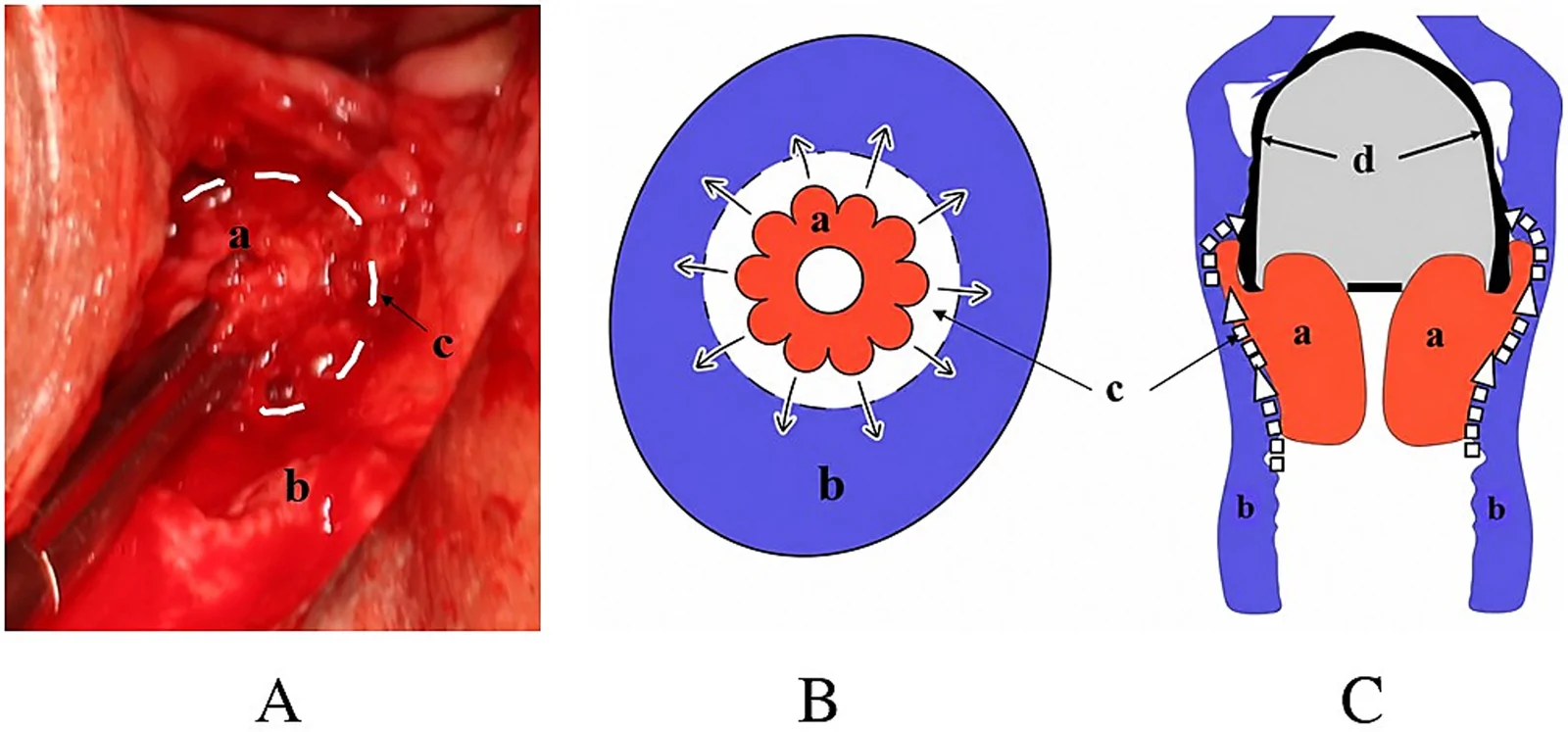
Dissection between the scar-related stenotic segment and the normal vaginal wall; (A) intraoperative view showing dissection between the scarred stenotic segment and the normal vaginal wall; the boundary between these tissues was incised, and dissection was advanced toward the foreign body; (B) schematic illustration of the dissection plane; the arrows indicate separation of the firm scar tissue from the surrounding normal vaginal wall; (C) coronal schematic view showing the anatomical relationship between the stenotic segment and the foreign body; the arrow indicates advancement through the plane between the scar tissue and the normal vaginal wall toward the foreign body; (a) scar tissue; (b) normal vaginal wall; (c) dissection plane between the scar tissue and the normal vaginal wall; (d) foreign body.

We expected to identify the foreign body at this point, but it was still not visible, even after an incision of ~1 cm. We realized that the firm induration did not represent the foreign body itself, but thick scar tissue extending deeper into the vagina (Fig. 3B). The dissection was therefore gradually extended. When the longitudinal incision reached ~3 cm, purulent material was released, indicating entry into the vaginal lumen beyond the stenosis, where part of the plastic foreign body became visible. We grasped the exposed end with a Kocher clamp and carefully dissected the adherent scar tissue around the foreign body to expose its entire circumference. The foreign body was then removed (Fig. 5). After confirming the absence of bladder and rectal injury, the incised vaginal wall and perineum were repaired with sutures. To prevent re-adhesion, a Penrose drain was placed in the vagina for 5 days. The patient remained free of complications during the 2-year follow-up period.

**Figure 5:**
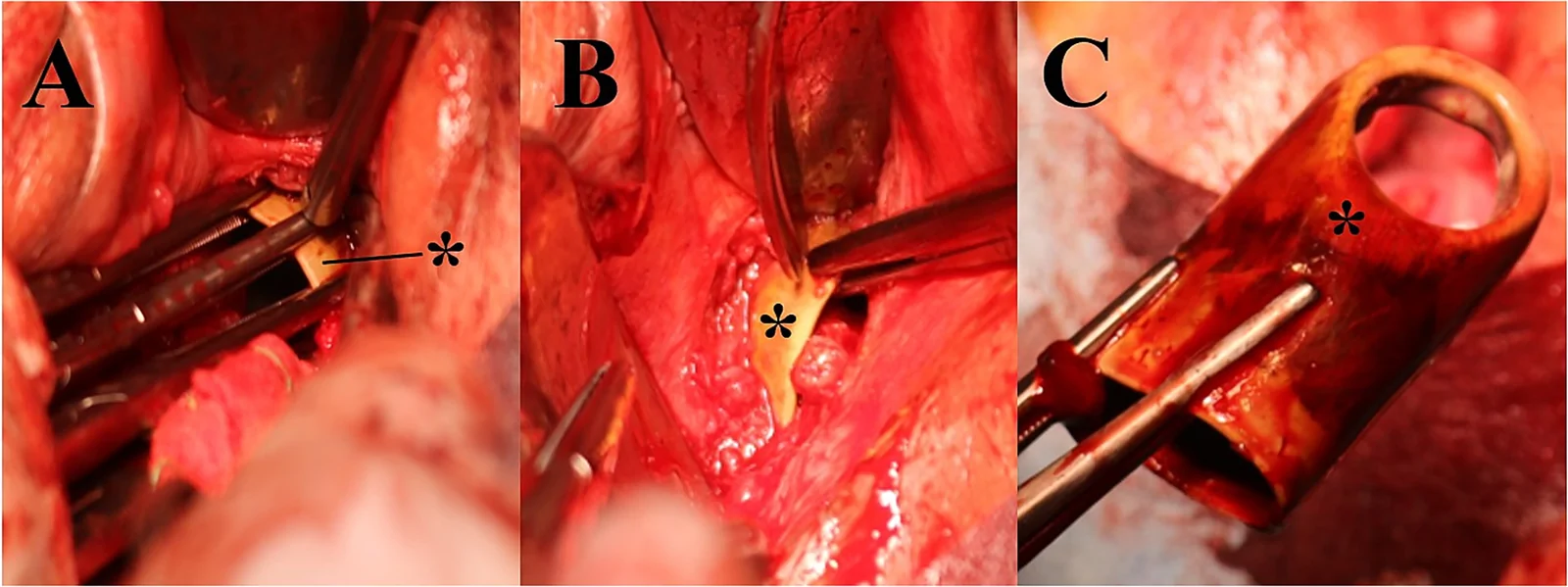
Removal of the vaginal foreign body; (A) the exposed end of the foreign body was grasped with a Kocher clamp; (B) scar tissue adherent to the foreign body was dissected away, and the foreign body was removed; (C) the removed foreign body is shown; the asterisk (*) indicates the foreign body.

## Discussion

Long-term retention of vaginal foreign bodies can cause vaginal stenosis [2, 3], but there are few reports that describe surgical techniques when removal is difficult.

In this case, the primary factor complicating removal was the difficulty in preoperatively recognizing the extent of vaginal stenosis. The palpable induration was initially assumed to represent the foreign body itself, but it was intraoperatively identified as actually being thick and extensive scar tissue, and the foreign body was located deeper than anticipated. It remained unclear how far the dissection and incision needed to be advanced, so the procedure was continued gradually and cautiously until the foreign body was identified. Retrospective review of preoperative MRI images did not allow accurate estimation of the thickness of the stenotic segment. This case highlights that long-retained vaginal foreign bodies may be associated with extensive, firm scar tissue and significant vaginal stenosis, and that palpable induration does not necessarily indicate the location of the foreign body itself.

Another important consideration was the risk of bladder and rectal injury during surgery, particularly because the boundary between the scar tissue and the vaginal wall was indistinct. Indigo carmine was instilled into the bladder to allow recognition of bladder injury. To minimize rectal injury, a perineal incision extending into the vaginal wall was made to facilitate confirmation of the anatomical relationship between the rectum and the stenotic segment and to provide access for rectovaginal dissection if necessary. These strategies enabled removal of the foreign body without injury to adjacent organs.

In cases of long-retained vaginal foreign bodies, unexpectedly thick scar tissue may develop, making localization of and access to the foreign body difficult. Careful surgical planning that anticipates these challenges is important.
